# Hepatitis B infection is highly prevalent among patients presenting with jaundice in Kenya

**DOI:** 10.1186/s12879-016-1409-2

**Published:** 2016-03-01

**Authors:** Missiani Ochwoto, James H. Kimotho, Julius Oyugi, Fredrick Okoth, Henry Kioko, Simeon Mining, Nancy L. M. Budambula, Elizabeth Giles, Anton Andonov, Elijah Songok, Carla Osiowy

**Affiliations:** Kenya Medical Research Institute (KEMRI), Nairobi, Kenya; Medical Microbiology Department, University of Nairobi, Nairobi, Kenya; Kenyatta National Hospital, Nairobi, Kenya; Moi University and Moi Teaching and Referral Hospital, Eldoret, Kenya; Jomo Kenyatta University of Agriculture and Technology, Nairobi, Kenya; National Microbiology Laboratory, Public Health Agency of Canada, Winnipeg, Canada; Present address: Embu University College, Embu, Kenya

**Keywords:** Genotype, HAV, HBV, HCV, HDV, HEV, Mutant, Kenya

## Abstract

**Background:**

Viral hepatitis is a major concern worldwide, with hepatitis A (HAV) and E (HEV) viruses showing sporadic outbreaks while hepatitis B (HBV) and C (HCV) viruses are associated with chronic hepatitis, cirrhosis and hepatocellular carcinoma. The present study determined the proportion, geographic distribution and molecular characterization of hepatitis viruses among patients seeking medical services at hospitals throughout Kenya.

**Methods:**

Patients presenting with jaundice at four selected hospitals were recruited (*n =* 389). Sera were tested for the presence of antibody to hepatitis viruses A through E, and HBV surface antigen (HBsAg). Nucleic acid from anti-HAV IgM antibody and HBsAg positive samples was extracted, amplified and sequenced.

**Results:**

Chronic HBV infection was the leading cause of morbidity among patients with symptoms of liver disease seeking medical help. Incident HCV, HEV and HDV infection were not detected among the patients in this study, while the proportion of acute HAV was low; HAV IgM positivity was observed in 6.3 % of patients and sequencing revealed that all cases belonged to genotype 1B. HCV seropositivity upon initial screening was 3.9 % but none were confirmed positive by a supplementary immunoblot assay. There was no serological evidence of HDV and acute HEV infection (anti-HEV IgM). HBsAg was found in 50.6 % of the patients and 2.3 % were positive for IgM antibody to the core protein, indicating probable acute infection. HBV genotype A was predominant (90.3 %) followed by D (9.7 %) among HBV DNA positive specimens. Full genome analysis showed HBV/D isolates having similarity to both D4 and D6 subgenotypes and D/E recombinant reference sequences. Two recombinant sequences demonstrated > 4 % nucleotide divergence from other previously known D/E recombinants.

**Conclusions:**

HBV is highly prevalent among patients seeking care for symptoms consistent with hepatitis, compared to the general population. Molecular characterization of HBV isolates indicated recombinant strains that may give rise to new circulating variants. There is a need to document the prevalence, clinical manifestation and distribution of the variants observed. HAV genotype 1B, prevalent in Africa, was observed; however, the absence of HCV, HDV and acute HEV in this study does not rule out their presence in Kenya.

## Background

Viral hepatitis is a serious public health problem worldwide. There are five main hepatitis-causing viruses designated A-E. Hepatitis A and E viruses (HAV, HEV) are often asymptomatic but can also result in acute disease often characterized by jaundice. They are transmitted primarily through a fecal oral route and are common in areas with sanitation challenges. HEV is responsible for sporadic hepatitis epidemics in Kenyan refugee camps [1]. Hepatitis B, C, and D viruses (HBV, HCV, and HDV) are transmitted mainly through body fluids, and often result in chronic liver infection. HBV is genetically classified into ten genotypes (A-J), most having subgenotype groupings, based on full genome divergence of >4.0 % but <7.5 %. HBV genotypes and subgenotypes have a global geographic distribution pattern [2]. Subgenotype A1 is predominant in Southern, Central and Eastern Africa [3–7]. Quasi subgenotype HBV/A3, previously HBV/A3, A4, and A5 [2], is found in West Africa and Haiti [8, 9]. Genotype D predominates in South America, the Mediterranean region and Eurasia [10]. Current systematic analysis of genotype D subgenotypes suggests that there are six subgenotypes, designated D1-D6, with several genotype D recombinants observed throughout Asia and Africa [11, 12]. HBV genotype E (HBV/E) is confined to West, Central and North Eastern Africa [5, 13]. It has low genetic diversity with no identified subgenotypes but it is often associated with recombinant variants including genotypes D and A. Kenya is a large country at the geographical junction of the distribution of the three HBV genotypes A, D and E; however, little is known about the molecular diversity of HBV in Kenya apart from analysis of the basal core promoter/precore (BCP/PC) and partial preS2/S regions [6, 7, 14].

HCV prevalence in Kenya has been reported to be approximately <1 % to 4.4 % in blood donors and patients attending clinics, respectively [15]; however, these data are based only on screening without further confirmatory testing by PCR or immunoblot assays. This prevalence is much lower than that reported among HIV-infected patients (6 %) and injection drug users (22 %) [16, 17].

In general, there is limited updated seroprevalence data on HAV, HEV, HDV, confirmed HCV and their disease burden in Kenya. The present study aimed to determine the prevalence and molecular characteristics of viral hepatitis A through E among patients with jaundice and other symptoms of liver disease in selected hospitals in Kenya.

## Methods

### Patient selection

Serum samples were obtained from 389 patients presenting with jaundice at four selected hospitals in Kenya. The hospitals were Kenyatta National Hospital (Nairobi), Moi Teaching and Referral Hospital (Eldoret), New Nyanza Provincial General Hospital (Kisumu), and Coast General Hospital (Mombasa). The hospitals were selected based on the high number of attendees in that region. Patients were enrolled prospectively from January-2012 to April-2013, based on their presentation of jaundice at the four hospitals, with no previous diagnosis of viral hepatitis based on patient recollection and hospital records. The study was designed to be a seroprevalence study performed on samples received in these hospitals for investigation of suspected viral hepatitis, such that all patients presenting with an observable jaundice condition, regardless of time of onset and duration, within the given study period were eligible for inclusion. At each hospital, individuals were triaged for suspected viral hepatitis following presentation of jaundice and in some cases other symptoms consistent with liver involvement such as abdominal tenderness or swelling, according to national guidelines for treatment of Hepatitis B and C virus infections [18]. Prior to any hospital testing, 4 mL whole blood was collected from study participants together with age and gender data. Following whole blood processing, approximately 1 mL serum was available for serological and molecular testing. Due to resource limitations and the requirement for test or procedure fees from patients [19], biochemical marker (ALT or AST) test results were largely not available for this patient subset or could not be accessed thereafter due to a lack of matching nominal data. The study population included individuals aged 15 years or above, thus, the only exclusion criteria was age <15 years. The minimum dataset provided with each specimen was gender and age.

### Ethical approval and consent

Ethical approval was obtained from the Kenya Medical Research Institute’s National Ethical Review Committee, approval number SSC 2436. Further approval was given by each participant or guardian through a signed consent form prior to drawing a blood sample and obtaining age and gender information.

### Viral hepatitis screening methods

The 389 specimens collected were simultaneously tested in different batches for HCV and HEV, followed by HAV testing, HBV testing and finally HDV testing of HBsAg positive specimens, based on our priority interests to obtain prevalence information for hepatitis viruses in Kenya that were lacking in the literature. Thus, antibody-confirmed HCV and HEV prevalence were a top priority, followed by HAV and lastly HBV/HDV. HAV and HBV were given lower priority as there are several high-quality literature references describing HAV and HBV prevalence among different Kenyan populations.

### Serological and molecular analysis

#### Hepatitis A virus (HAV)

Sera were tested for the presence of IgM antibody to HAV using the International Immunodiagnostics HAV-IgM Ab EIA kit (International Immunodiagnostics Inc., California, USA). Nucleic acid was extracted from all samples that were screened positive for HAV antibody. HAV RNA was extracted using the NucliSENS easy MAG total nucleic acid automated extraction system (BioMerieux, Quebec, Canada). HAV amplification was performed by nested PCR using primers listed in Table 1 in a One Step RT-PCR reaction. The PCR mixture contained 1.75U Expand High Fidelity polymerase (Roche Diagnostics), 250 μM dNTPs (Thermo Fisher Scientific), 20 μM reverse and forward primers, 1X High Fidelity buffer, and 5 μl of the extracted RNA in a total volume of 50 μl. After the first round, 5 μl was used for the 2nd round nested reaction using a PCR mixture as above with nested primers (F2/R2). The primers were based on M14707 Hepatitis A wild type sequence [20].

**Table 1:** Primers used for amplification of HAV and HBV

Virus to be amplified	Primer Name	Sequence (5′–3′)
HAV^a^	HAV F1	GACAGATTCTACATTTGGATTGGT
	HAV R1	CCATTTCAAGAGTCCACACACT
	nested HAV F2	CTATTCAGATTGCAAATTACAAT
	nested HAV R2	AACTTCATTATTTCATGCTCCT
HBV^b^	S1f	TCCTGCTGGTGGCTCCAG
	S1r	CGTTGACATACTTTCCAATCAA
	nested S2f	ACCCTGYRCCGAACATGGA
	nested S2r	CAACTCCCAATTACATARCCCA
	C1 (EP1)	GCATGGAGACCACCGTGAAC
	C1 (EP2)	GGAAAGAAGTCAGAAGGCAA
	nested C2 (EP3)	CATAAGAGGACTCTTGGACT
	nested C2 (EP4)	GGCAAAAAAGAGAGTAACTC

^a^[20]; ^b^[53, 54]

#### Hepatitis B virus (HBV)

Hepatitis B surface antigen and antibodies to Hepatitis B core antigen were analyzed by electrochemiluminescence EIA using the COBAS e411 platform (Elecsys; Roche Diagnostics, Quebec, Canada). Nucleic acid was extracted from all samples that were screened positive for HBsAg. HBV DNA from HBsAg positive samples was extracted using the QIAamp® DNA blood mini kit (Qiagen Inc., Ontario, Canada). Two hundred microliters (200 μl) of the sample was extracted as instructed in the user manual. The extract was eluted in 60 μl nuclease free water (Ambion®, Thermo Fisher Scientific, Massachusetts, USA). Five microliters (5 μl) of the HBV extract was amplified in a nested PCR. Two different sets of primers were used to target the HBsAg (S1) and BCP/PC region (EP1-2; Table 1) in a total volume of 50 μl per tube. Each tube contained buffers, 20 μM forward and reverse primers, 2.5U Amplitaq Gold polymerase (Thermo Fisher Scientific) and 1.25 mM dNTPs. Full length HBV genome analysis was carried out by amplification using published primers [21]. The PCR reaction conditions for both the HBsAg and BCP/PC regions were 94 °C for 10 min, 40 cycles of 94 °C for 30 s, 55 °C for 30 s and 72 °C for 40 s and final extension of 72 °C for 5 min. The amplicons were viewed following electrophoresis on a 2 % agarose gel and any PCR negative sample was re-amplified by nested PCR. Five micro-liters of the 1st stage PCR amplicons were used for the second amplification using S2 and C2 primers for HBsAg and BCP/PC, respectively. The master mix and the PCR profile were similar to the first round. All 1st and 2nd round amplicons were gel-purified prior to sequencing and the sequences were treated as previously described by Kowalec *et al.,* [21].

#### Hepatitis C virus (HCV)

Antibodies to HCV were measured using the VITROS ECiQ platform (Ortho Clinical Diagnostics, Ontario, Canada) with confirmation of positive results performed using the INNO LIA HCV Score assay (Fujirebio, Ghent, Belgium) and HCV Real-time PCR. Nucleic acid was extracted from all samples that were initially screened positive for HCV antibody. HCV RNA was extracted using the NucliSENS easy MAG total nucleic acid automated extraction system (BioMerieux, Quebec, Canada). HCV Real-time PCR was performed as previously described [22]***.***

#### Hepatitis D virus (HDV)

Antibodies to HDV were tested using the International Immunodiagnostics HDV Ab EIA kit (International Immunodiagnostics Inc., California, USA).

#### Hepatitis E virus (HEV)

Sera were tested for the presence of IgG and IgM antibodies to HEV using commercial ELISA assays (Wantai, Beijing, China).

### Sequencing the isolates

All PCR positive amplicons were purified using the Qiagen Gel purification kit according to the manufacturers recommended protocol. Purified DNA was quantified with Nanodrop (Thermo Fisher Scientific), and purified DNA (50 ng) was then sequenced using an automated ABI 3750 XL Genetic Analyzer (Thermo Fisher Scientific). Amplification primers (Table 1) were used for sequencing. Directly amplified sequences were assembled and analysed using DNA sequence analysis software (Lasergene software suite v7.1.0, DNASTAR). Sequences were aligned and edited separately using ClustalX v2.0.1 [23] and Bio-Edit software [24], respectively.

### Phylogenetic analysis

Maximum Likelihood phylogenetic analyses of the HBV surface antigen-coding region (681 bp; nt 155–835) and the HBV BCP/PC region (307 bp; nt 1653–1959) were performed using the Kimura 2-parameter + γ substitution model as the most appropriate model, and neighbor-joining tree construction with 500 bootstrap replicates was performed by MEGA v.5.2 [25].

Full length HBV genome phylogenetic analysis was performed by the GTR + γ + I model as described above. Nucleotide pairwise distance of HBV genotype D full genome sequences was measured using DIVEIN software by the GTR + γ + I substitution model [26]. Mutations were analyzed in the BCP/PC and HBsAg surface regions by alignment with genotype reference sequences.

Intergenotypic recombination analysis of suspect recombinant sequences was performed using SSE v1.1 [27] with a set of 295 GenBank reference sequences (available upon request).

HBV full genome and HAV partial genome sequences obtained in this study were submitted to the National Center for Biotechnology Information GenBank database [KP168416 to KP168435; KT723433 to KT723437].

### Statistical analysis

The data was analyzed using statistical software (SPSS v.16.0). Fisher’s exact test was used to analyze associations between age and geographical region among HBV genotypes, the 2-tailed Z-test at 95 % significance was used to compare HAV prevalence among regions, and the Student’s *T* test was used to analyze differences in mean age based on HBsAg positivity. A P value of <0.05 was considered significant.

## Results

### Patient population demographic profile

**Fig. 1:**
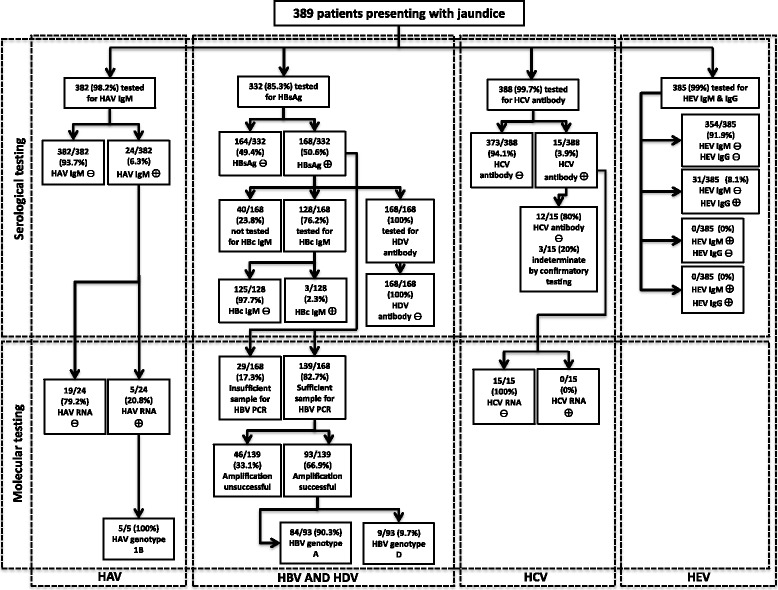
A flow-chart detailing the viral hepatitis serological screening and molecular testing algorithm for specimens collected from individuals presenting with jaundice at four Kenyan hospitals. The associated results for each group of specimens for each hepatitis virus are also shown

Three hundred and eighty nine (389) blood samples were collected from patients presenting with jaundice at four selected hospitals in Kenya. These samples include 245 from Nairobi city and its surroundings (KN), 76 samples from Kisumu (KSM), 40 samples from Mombasa (MBS) and 28 from Eldoret (ELD). Screening for HCV antibody was conducted on 388 samples, while 385, 382, and 332 samples were screened for HEV, HAV and HBV, respectively, as sample volumes became exhausted. Figure 1 illustrates the testing performed for each hepatitis virus among the study specimens and the subsequent results as a flow chart. The mean age (± SD) of patients from each region was as follows; KN 39.8 years (±14.1), KSM 35.2 years (±11.9), MBS 36.2 years (±12.3), ELD 32.1 years (±10.4). The male to female ratio was 4:3. Table 2 shows the prevalence of each detectable hepatitis virus according to gender and region, as well as the mean age (± SD) and overall prevalence ± 95 % confidence intervals (CI) and proportion for each detectable hepatitis virus

**Table 2:** Mean age and prevalence of viral hepatitis according to gender and region^a^

		HAV	HBV	HEV
Age; mean ± SD (range)		35.1 ± 15.3 (16–84)	36.5 ± 11.2 (16–64)	41.4 ± 14.5 (17–75)
Gender; % (proportion)	Male; *n =* 218	66.7 % (16/24)	65.5 % (110/168)	45.2 % (14/31)
	Female; *n =* 171	33.3 % (8/24)	34.5 % (58/168)	54.8 % (17/31)
Region^b^; % (proportion)	KN; *n =* 245	6.3 % (15/238)	33.8 % (74/219)	12.9 % (31/241)
	KSM; *n =* 76	9.2 % (7/76)	79.8 % (59/74)	0 % (0/76)
	MBS; *n =* 40	5.0 % (2/40)	81.8 % (9/11)	0 % (0/40)
	ELD; *n =* 28	0 % (0/28)	92.9 % (26/28)	0 % (0/28)
Overall Prevalence ± 95 % CI (proportion)		6.3 % ± 2.43 % (24/382)	50.6 % ± 5.38 % (168/332)	8.1 % ± 2.72 % (31/385)

^a^The overall prevalence of HCV (*n =* 388) and HDV (*n =* 168 HBsAg positive samples) was determined to be 0 %

^b^Region codes are described in the Results

#### HAV

Twenty-four out of 382 (6.3 % ± 2.43 %, 95 % CI) samples were anti-HAV IgM positive. Based on their area of residence, most incident cases were from Kisumu (7/76 = 9.2 %), followed by Nairobi (15/238 = 6.3 %) and Mombasa (2/40 = 5.0 %); however, no significant prevalence differences were observed between regions (*p* > 0.05). None of the 28 patients from Eldoret had evidence of acute HAV infection. The majority of infected persons were 20–49 years of age, with a prevalence of 6.5 % ± 2.83 %, 95 % CI. Stratified age group analysis showed that most affected males were in the 20–29 year age group (43.8 %) while females were most affected in the 30–39 year age group (37.5 %). Most cases in the cities of Nairobi and Mombasa belonged to the 20–29 year age group while in Kisumu most of those infected were 40–49 years of age. Five patients (1.3 %) were found to have HAV/HBV co-infection; three from Kisumu, one from Nairobi and the other from Mombasa. Five of 24 samples were RNA positive. Phylogenetic results from these five isolates revealed that they were all genotype 1B [GenBank: KT723433 to KT723437].

#### HBV

Screening for hepatitis B virus surface antigen (HBsAg) was performed with 332 individual samples from the 4 clinic sites. Out of the 332 samples, 168 were HBsAg positive (50.6 % ± 5.38 %, 95 % CI). IgM antibody to the core protein (anti-HBc IgM) was tested in 128 out of the 168 HBsAg positive of which 3 (2.3 %) were positive, indicating possible acute infection or acute exacerbation of chronic hepatitis. Based on the area of residence, Eldoret had the highest proportion of HBsAg positive cases, 26/28 (92.9 %), followed by Mombasa, 9/11 (81.8 %), Kisumu, 59/74 (79.8 %), and Nairobi, 74/219 (33.8 %). The mean age (± SD, range) for those HBsAg positive was not significantly different (*p* = 0.053) from those HBsAg negative, at 36.5 years (±11.2, 16–64 years) vs. 39.4 years (±15.7, 15–85 years), respectively. A male to female ratio of 2:1 was observed for HBsAg positive individuals. One-hundred and thirty-nine HBsAg positive samples having sufficient volume for further testing were extracted for HBV DNA, with 93 (66.9 %) successfully amplified for either the HBV surface region (*N =* 86), and/or the BCP/PC region (*N =* 79). Twenty samples chosen at random were amplified for full genome sequencing. HBV genotype A predominated (90.3 %) followed by genotype D (9.7 %).

#### HCV

Initial screening of 388 samples for antibody to HCV by chemiluminescent immunoassay resulted in 15 initially reactive specimens (3.9 %). However, upon confirmatory testing by INNO-LIA HCV Score immunoblot assay, all specimens were negative for HCV antibody, with the exception of 3 indeterminate INNO-LIA results. None of the 15 samples were found to be positive for HCV RNA by Real-time PCR.

#### HDV

No anti-HDV antibodies were detected among the 168 HBsAg positive samples.

#### HEV

The prevalence of IgG antibodies to HEV among patient samples was 8.1 % ± 2.72 %, 95 % CI (31/385). None of the specimens were anti-HEV IgM positive. More females (54.8 % ± 17.52 %, 95 % CI) than males (45.2 % ± 17.52, 95 % CI) were exposed to HEV, based on IgG results.

### Phylogenetic analysis of HBV isolates

**Fig. 2:**
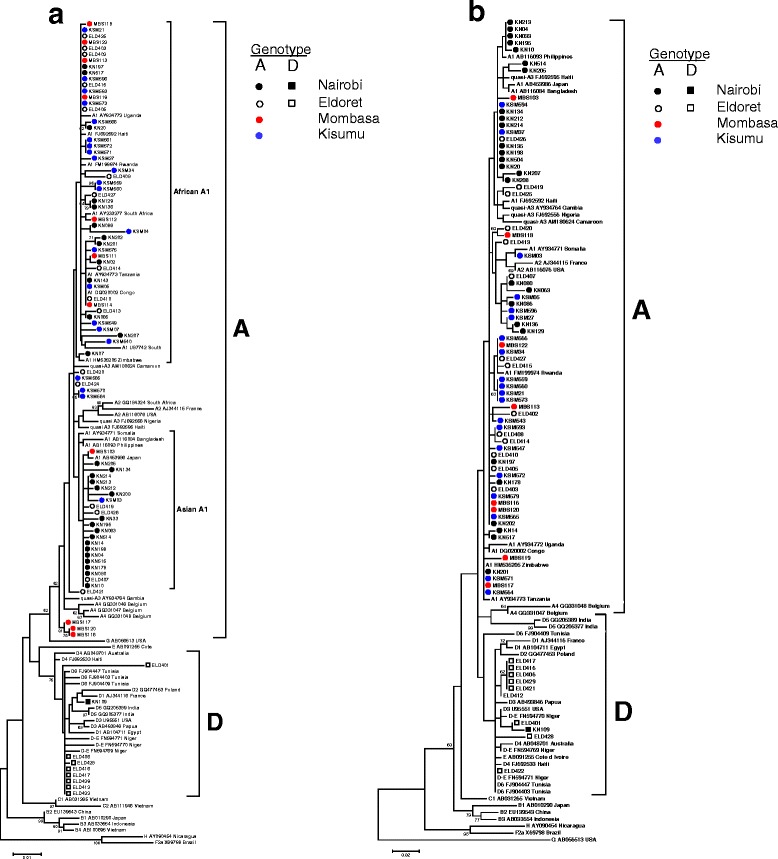
Phylogenetic analysis of Kenyan HBV subgenomic sequences. Specimen codes are described in the Results. Maximum likelihood analysis and neighbor-joining tree construction with 500 bootstrap replicates was performed by the K2 + γ model by MEGA v.5.2. Comparative GenBank sequences are designated by the subgenotype followed by the accession number and country of origin. Bootstrap confidence values ≥60 % are shown. **a** HBsAg (681 bp; nt 155 – 835) (**b**) BCP/PC region (307 bp; nt 1653–1959)

Phylogenetic analysis of partial genome sequences showed that HBV genotype A isolates clustered with sub-genotype A1 reference sequences with a distinction noted between the Asian A1 clade and the African A1 clade for HBsAg region analysis. As shown in Fig. 2, isolates from all four sites cluster with sequences from neighboring Tanzania, Uganda, Rwanda, Congo, Zimbabwe and South Africa (African A1 clade). The majority of isolates (>70.0 %) from Nairobi and its environs clustered with Asian A1 sequences from the Philippines, Bangladesh, Somalia and Japan (Fig. 2a). Based on HBsAg gene analysis, three genotype A sequences from Mombasa (MBS116, MBS117, and MBS120) formed a distinct sub-cluster supported by 62 % bootstrapping for 500 replicates by Maximum Likelihood phylogenetic analysis.

Compared to HBV/A isolates, there was a statistically significant difference observed in age and geographical region with HBV/D isolates (Fisher test, *p =* 0.02). Patients with genotype D (8/9) were mainly from the region of western Kenya in Eldoret, while 1/9 genotype D specimens was observed in Nairobi. All genotype D-infected individuals were male with a mean age of 43.0 ± 2.0 years whereas, individuals infected with genotype A had a mean age of 34.0 ± 4.0 years.

**Fig. 3:**
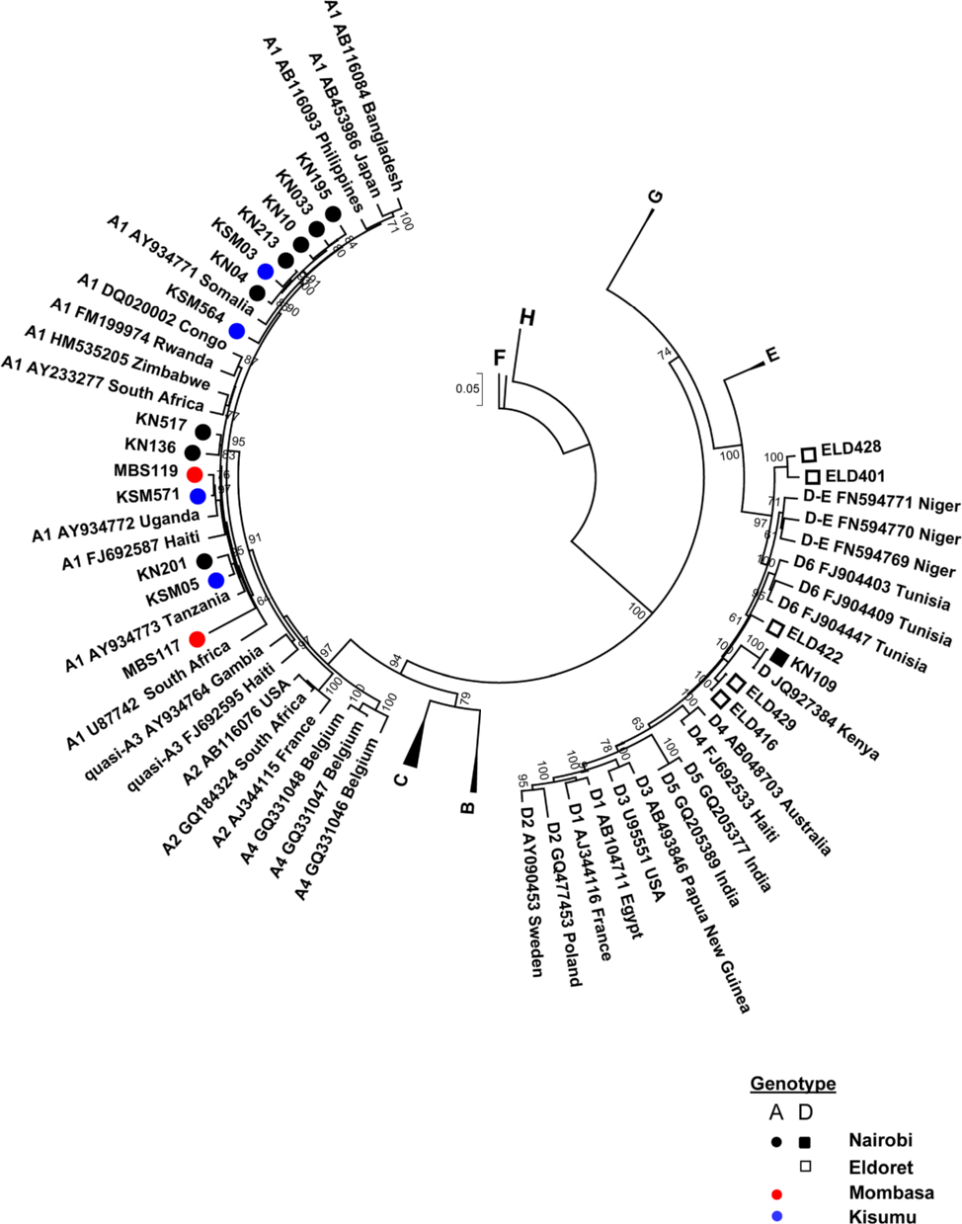
Phylogenetic analysis of 20 Kenyan complete HBV genome sequences. Specimen codes are described in the Results. Maximum likelihood analysis and neighbor-joining tree construction with 500 bootstrap replicates was performed by the GTR + ϒ + I model by MEGA v.5.2. Comparative GenBank sequences are designated by the subgenotype followed by the accession number and country of origin. Bootstrap confidence values ≥60 % are shown. The ruler shows the branch length for a pairwise distance equal to 0.05. The GenBank accession numbers for the sequences reported are KP168416 to KP168435

Fourteen genotype A and six genotype D samples were analyzed for full genome sequence in order to clarify subgenotype determination (Fig. 3). The mean nucleotide distance within the 14 genotype A full genome sequences was 3.47 % ± 0.25 %, owing to the unusual sequence of isolate MBS117; if excluded the mean distance is 2.71 % ±0.23 %. MBS117 had sequence characteristic of genotype A; however, sequence alignment with all reference genotypes showed that a region spanning approximately nucleotides 2860 to 2953 within the PreS1 region had nucleotide substitutions characteristic of genotype E, including the 3 nucleotide deletion leading to loss of PreS1 amino acid 11.

Based on full genome phylogenetic analysis, four genotype D isolates, ELD416, ELD422, ELD429, and KN109, clustered apart from reference HBV genotype D subgenotypes, except GenBank accession number JQ927384, a recently submitted genotype D6 strain from Kenya (Fig. 3). ELD401 and ELD428 were also observed to cluster separately from all other genotype D subgenotypes. Nucleotide distance measurements suggested that ELD416, ELD422, and ELD429 had similar identity to subgenotypes D4 and D6, but also D-E recombinant reference sequences, compared to other subgenotypes of D, while KN109 had a nucleotide distance identity falling within reference subgenotype D6 sequences (Table 3). All genotype D isolates had the signature amino acid rt237T, characteristic of D6 and D/E recombinant sequences [11]. Full genome sequence analysis of ELD401 and ELD428 demonstrated the lack of the genotype D-specific 33 nt deletion following the PreS1 start codon, similar to the observation of Chekaraou *et al.* [28]. ELD401 and ELD428 full genome sequences had a nucleotide distance >4 % from all HBV/D subgenotypes and D/E recombinant reference sequences, which is the minimum requirement for a new subgenotype [2, 29].

**Table 3:** Mean pairwise nucleotide distances between genotype D subgenotypes and Kenyan complete genome genotype D sequences

		Genotype D subgenotype^a^						
		D1	D2	D3	D4	D5	D6	D-E
Genotype D full genome sequences	ELD401	**0.0612**	**0.0648**	**0.0613**	**0.0534**	**0.0688**	**0.0494**	**0.0488**
	ELD416	**0.0405**	**0.0443**	0.0382	0.0315	**0.0467**	0.0302	0.0329
	ELD422	**0.0430**	**0.0481**	**0.0425**	0.0332	**0.0512**	0.0317	0.0333
	ELD428	**0.0543**	**0.0577**	**0.0535**	**0.0452**	**0.0612**	**0.0412**	**0.0423**
	ELD429	**0.0412**	**0.0450**	0.0389	0.0322	**0.0474**	0.0308	0.0335
	KN109	**0.0505**	**0.0534**	**0.0514**	**0.0445**	**0.0586**	0.0389	**0.0467**

^a^GenBank reference sequences used were as follows: D1 (AF280817, AJ344116, AF151735, GU456684, AB104711), D2 (AY090453, X72702, GQ477453, JF754597, AB078032), D3 (U95551, AY233296, AJ627217, EU921419, AB493846), D4 (HQ700500, AB033559, AB048703, FJ692533, HE974378), D5 (GQ205377, GQ205385, GQ205389, AB033558, GQ205382), D6 (FJ904409, FJ904447, FJ904403, FJ904433, FJ904438, FJ904410, FJ904395, FJ904442, KF170740, JQ927384), D-E recombinant (FN594771, FN594769, FN594770); values in bold represent nucleotide diversity >4 %

### HBV recombination analysis

**Fig. 4:**
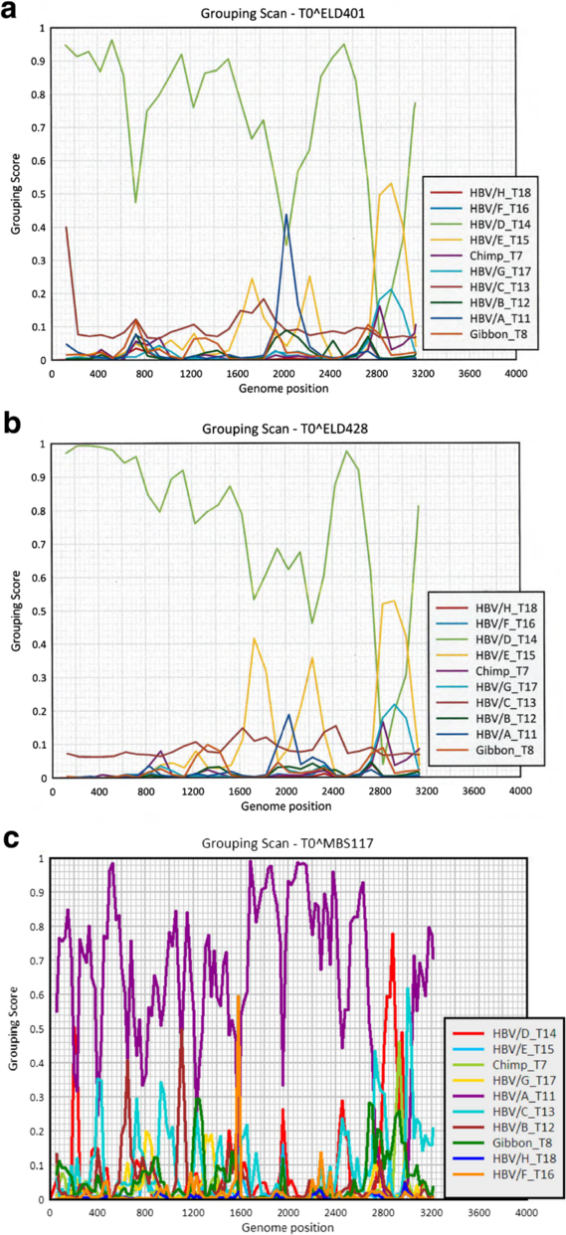
Grouping Scan analysis of the complete genome of (**a**) ELD401 (**b**) ELD428 (**c**) MBS117. The complete genome was scanned against GenBank reference sequences of human HBV genotypes A-H and chimpanzee- and gibbon-derived strains (*n =* 295 sequences). Association values ≥0.5 indicate phylogenetic clustering with the specified genotype reference group, while those <0.5 indicate an outgroup position having no phylogenetic association with the genotype reference groups

As genotype D complete genome sequences did not readily cluster with a specific subgenotype, or were found to have a nucleotide distance >4 % from other subgenotypes, recombination analysis was conducted using the Simmonic software package (SSE v1.1). Recombination analysis revealed that isolates ELD401 and ELD428 were putative D/E recombinants (Fig. 4a and b) with ELD401 also demonstrating a grouping scan value approaching 50 % for genotype A recombination at nucleotide positions 1840 to 2240 (Fig. 4a). Genotype E recombination was observed within the preS1 region (nt 2800–3000) with both sequences, similar to previously described D/E recombinant isolates [28], although both ELD401 and ELD428 had >4 % nucleotide distance from recombinant sequences deposited in GenBank [FN594769, FN594770, FN594771]. Recombination was also investigated with ELD416, ELD422, ELD429 and KN109 full genome sequences, but all were determined to be genotype D sequences without evidence of recombination. The grouping scan analysis of genotype A specimen MBS117 showing putative recombination with genotypes D and E is shown in Fig. 4(c).

### Profiling mutations in the HBV genome

#### BCP/PC region substitutions

A total of 79 DNA positive samples had sequence available to analyze substitutions within the BCP/PC region (70 genotype A and 9 genotype D sequences). The percentage of select mutations within this region is shown in Table 4. Five genotype A isolates either had a 1 nt deletion mutation at nucleotide 1825, a 1 nt insertion at nucleotide 1838, or a 13 nt insertion at nucleotide 1915. The precore stop codon mutation, A1896, was identified in 4.3 % (3/70) genotype A samples and 44 % (4/9) of genotype D samples. A1896 co-existed with T1858 in genotype A sequences, and all HBV/D strains were found to have T1858. The double mutation T1762/A1764 was observed with both genotype A and D sequences. A total of 23 (33 %) HBV/A and 4 (44 %) HBV/D isolates had the double BCP mutation or a variation of it (T1762/G1764 or A1762/A1764).

**Table 4:** Prevalence of HBsAg and BCP/PC mutations observed among HBV genotype A and D sequences

	Mutation (%)^a^	
Nucleotide mutation	Genotype A	Genotype D
HBsAg amino acid substitutions^b^	11/77 (14)	0
BCP/PC insertion/deletions^c^	5/70 (7)	0
C1653T	2/70 (3)	3/9 (33)
A1752C	0	1/9 (11)
T1753V	3/70 (4)	2/9 (22)
A1762T/G1764A^d^	23/70 (33)	4/9 (44)
G1809T	65/70 (93)	0
A1811T/C	7/70 (10)	0
C1812T	62/70 (89)	0
C1858T	4/70 (6)	9/9 (100)
G1862T/C	24/70 (34)	0
G1888A	47/70 (67)	0
G1896A	3/70 (4)	4/9 (44)

^a^Genotype A: 77 and 70 samples could be amplified for HBsAg and BCP/PC sequences, respectively; Genotype D: Sequence was available in both regions for all 9 samples. ^b^The following substitutions within the antigenic determinant region (amino acids 100–160) of the HBsAg gene were observed: M103I, L109P, I110L, G112R, T114S, T115A, T118K, Q129R, G130N, M133I, F134I/V, P135H, T143M, S155R; ^c^see text; ^d^includes A1762/A1764 (6 genotype A and 2 genotype D) and T1762/G1764 (3 genotype A)

The substitutions characteristic of subgenotype A1 [4] were highly prevalent among study isolates. The G1809T, A1811T/C, and C1812T substitutions were observed in 93 %, 10 %, and 89 % of genotype A sequences respectively, with 5 of 7 sequences having a triple nt 1809–1812 mutation also having the T1762/A1764 mutation or a variation of it. G1862T/C and G1888A mutations were observed in 34 % and 67 % of genotype A sequences, respectively (Table 4).

#### HBsAg-coding region substitutions

A number of substitutions were observed in the antigenic determinant region (amino acids 100–160) of the HBsAg coding sequence from genotype A isolates; no HBsAg substitutions were observed with genotype D sequences (Table 4). Three isolates (KN202, KN207 and KN134) had the mutation sM133I. Three different substitutions (sI110L, sG112R, and sT114S) were observed in two different isolates each. Two isolates (KSM561, KSM572) from the same geographic area, had two substitutions (sG112R, and sT114S). One isolate, KSM04, had four substitutions (sM103I, sQ129R, sG130N, sP135H) within the antigenic determinant region.

## Discussion

This is the first publication describing the epidemiological and molecular characterization of viral hepatitis in Kenya in patients seeking medical care in different hospitals. HBsAg positivity was identified in half of the patients tested, with the majority having chronic infection and only 2.3 % presumably acutely infected based on the detection of anti-HBc IgM positivity. Acute HAV infection was detected in 6.3 % of tested patients; however, in the remaining patients, no laboratory markers of HCV, HDV or HEV infection were detected.

HAV is endemic in areas with sanitation challenges due to lack of control of transmission factors and presence of HAV in the environment [30, 31]. In our study, HAV was observed in cities faced with overpopulation and increases in high density substandard housing [32]. Lack of access to clean water and food could also be contributing factors for HAV exposure. HAV genotype 1B which was responsible for acute infection in the present study, is associated with water contamination [31], thus further investigation is required to determine if this is the main mode of transmission in the country.

Most of the previous studies on HAV in Kenya have focused on the high prevalence rates in children; 43.7 % [32] and 63.2 % [33]. Only one study, by Greenfield *et al.* [34], reported a prevalence of 12 % among adults. A number of studies have showed that by the age of 10 years, 90 % of children in areas where HAV is endemic have been infected with the virus [35, 36], thus older children and adults are generally immune. The prevalence observed in this study (6.3 %) was within the age group of adults seeking medical services which was unexpected; however, as sanitation improves in urban areas, there could be a shift in the age of HAV susceptible individuals from young children to adults [37]. HAV vaccine is available in leading hospitals in Kenya, although its uptake is still low.

There are few reports on HCV, HDV and HEV infection in Kenya. Initial anti-HCV screening in the present study indicated a prevalence of 3.9 %, comparable with previous reports of Ilako et al., [38] at 2.6 %, Karuru et al., [15] at 4.4 % and Harania et al., [39] at 1.0 %, none of which employed anti-HCV supplementary testing. Use of confirmatory testing in the present study showed that all suspect specimens were negative for HCV antibody, which may indicate that previously reported data may include false-positive results.

Previous reports on HDV prevalence in Kenya described a low prevalence in most parts except northern Kenya where the prevalence was 31 % [40]. Our study found no specimens positive for HDV antibody, even among samples from northern Kenya. This may be due to differences in testing methods among the studies, or it may be that rates of HDV infection have decreased over time in Kenya. However, such a comparison must be interpreted with caution, as although HBV prevalence in Kenya is high, the absence of HDV detection does not rule out its presence in the country.

To date there is limited data on the source and genotype of HEV in Kenya. Our study found no acute cases of HEV as indicated by IgM positivity. However, a prevalence of 8.1 % anti-HEV IgG positivity indicates that the virus is present in Kenya, albeit at a modest rate.

Kenya is considered endemic for hepatitis B infection (>8.0 % HBsAg prevalence; [36]), and case rates appear to be increasing [41]. Efforts to reduce transmission through infant immunization and blood donor screening have been promoted in the country; however, HBV infection remains high [16, 42]. The highest number of HBsAg positive cases were observed in western Kenya (Eldoret) at 92.9 % followed by Mombasa at 81.8 %, with other regions showing marked differences, such as 33.8 % HBsAg positivity in Nairobi, which was somewhat surprising. Bias may have been introduced in the HBsAg prevalence estimate for Mombasa, as only one quarter of specimens collected from the participating hospital were tested for HBsAg, resulting in a very high prevalence (81.8 %), which may not be representative of the region. Nevertheless, almost 90 % of specimens from the Kenyatta National Hospital in Nairobi, and 100 % of specimens collected from the Moi Teaching and Referral Hospital in Eldoret were tested for HBsAg positivity, with marked differences observed between the two sites (33.8 % vs. 92.9 %, respectively). As all patients were symptomatic with jaundice, the differences in causality may be due to differences in the patient population seeking medical attention among regions. For example, a previous investigation has shown a considerably higher prevalence of HBsAg positivity in Eldoret (17.8 %) compared to Nairobi (7.7 %) among pregnant women [43]. Alternatively, environmental or infectious agents other than viral hepatitis associated with jaundice may be involved, such as malaria and other parasitic infections. For example, although both Nairobi and Eldoret are located at higher altitudes having conditions thought to be unfavorable for malarial transmission, Nairobi has had recurrent epidemics in past decades [44] and appears to be presently in the midst of increasing malaria incidence, partly due to climate change [45, 46]. These past epidemics and the higher density urbanization of Nairobi may result in increased malarial diagnostic experience and resources relative to other regions of intermediate malaria prevalence, such as Eldoret [47]. Similarly, a report of improved diagnostic capability for malaria within an Eldoret clinic showed a significant decrease in the number of confirmed malaria cases [48].

Kenya is a country at the geographical junction of the distribution of the three common HBV genotypes in Africa, A, D and E, which provides an opportunity to study the diversity and interactions of these genotypes. In this study, we successfully sequenced 93 samples from patients attending 4 hospitals throughout Kenya. Genotype A was the major circulating genotype, followed by genotype D, which was primarily observed in western regions of Kenya. Earlier Kenyan studies had observed genotypes D and E, with both subgenotypes D4 and D6 in circulation among blood donor and liver patients, respectively [6, 7, 14]. In this study, several recombinant HBV D/E variants were observed in western Kenya, with recombination breakpoints falling within the preS coding region. Recombinants of HBV genotypes D and E have been described in Northern Africa [13, 28, 49]. The recombinant variants observed in the present study demonstrated nucleotide divergence >4.0 % compared to previously reported strains, suggestive of a new subgenotype. Recombinant strains may develop following HBV intergenotype dual infections [50] or may persist as a circulating strain throughout a population. The clinical manifestations of D/E recombinant strains are poorly documented. A genotype A variant having putative recombination with genotype E and/or D, collected from coastal Kenya, was also observed within the study population. There are very few reports of genotype A/E recombinants and therefore their impact needs to be assessed.

Based on full genome analysis, most genotype D sequences shared identity with subgenotypes D4 and D6; however, amino acid E161 of the polymerase terminal protein, characteristic of D6 sequences, was only observed with study sequences, as opposed to the subgenotype D4 signature V161 amino acid [11]. Similarly, all genotype A samples in the study having sufficient sequence information had amino acid signatures consistent with subgenotype A1 [4], and were found to have a low prevalence of HBsAg immune escape mutations. The BCP double mutation, T1762/A1764 was observed with both genotype A and D isolates. This mutation has been commonly associated with more severe clinical outcomes [51], although this study did not investigate the association with severity of liver disease.

This study has some limitations. The use of specimens from adults seeking medical care at major hospitals may possibly bias prevalence estimates towards a higher value. A further limitation is the possibility that patients chronically infected with HBV and having knowledge of their HBV status were inadvertently included in the study population, although every attempt was made to eliminate symptomatic patients presenting for HBV management. The lack of detection of acute viral hepatitis and/or viral RNA may have been affected by the timing of specimen collection relative to the date of symptom onset, if samples were collected during the post-viremic period. Similarly, cases of acute HCV infection may have been missed among the 373 patients testing negative for HCV antibody, as these samples were not further tested for HCV RNA. The low incidence of acute HBV infection was somewhat unexpected; however, this observation was likely due to some participants experiencing symptomatic flares of chronic infection, for which they were diagnosed for the first time. This observation may be related to variable healthcare seeking behavior, particularly regarding large hospital facilities, which has been described in Kenya [52].

## Conclusions

HBV is highly prevalent among patients seeking care at Kenyan hospitals for symptoms consistent with hepatitis, compared to the national HBV prevalence. Molecular characterization of circulating HBV indicated recombinant strains that may give rise to new circulating variants. There is a need to document the prevalence, clinical manifestation and distribution of the variants observed. HAV genotype 1B, prevalent in Africa, was observed within the study population. The absence of HCV, HDV and acute HEV in this study does not rule out their presence in Kenya.

## Availability of data and materials

The datasets supporting the conclusions of this article are available in the GenBank database repository [KP168416 to KP168435; KT723433 to KT723437].

## Abbreviations

HAV: hepatitis A virus
HBV: hepatitis B virus
HCV: hepatitis C virus
HDV: hepatitis D virus
HEV: hepatitis E virus
HBsAg: hepatitis B virus surface antigen
HBc: hepatitis B virus core antigen
BCP/PC: hepatitis B virus basal core promoter/precore genomic region
preS2/S: hepatitis B virus pre-surface/surface genomic region
ELD: Eldoret
KN: Nairobi and its environs
KSM: Kisumu
MBS: Mombasa
